# PTPRO represses breast cancer lung metastasis by inhibiting the JAK2-YAP axis

**DOI:** 10.1038/s41598-025-91341-0

**Published:** 2025-02-27

**Authors:** Xiao Xiong, Jingfang Liu, Xiaotong Wu, Zhimeng Yao, Yuhua Meng, Shuang Liu, Yexi Chen, Hongzheng Ren, Shegan Gao, Xiaofu Qiu, Hao Zhang

**Affiliations:** 1 Department of Urology, Guangdong Second Provincial General Hospital, Integrated Chinese and Western Medicine Postdoctoral Research Station, School of Medicine, Jinan University, Guangzhou, China; 2 Institute of Precision Cancer Medicine and Pathology, School of Medicine, Jinan University, Guangzhou, China; 3 Department of Urology, The First Affiliated Hospital of Jinan University, Jinan University, Guangzhou, China; 4 Department of Hematology, Guangdong Second Provincial General Hospital, Guangzhou, China; 5 Department of General Surgery, The Second Affiliated Hospital of Shantou University Medical College, Shantou, China; 6 Department of Pathology, Gongli Hospital, Naval Medical University, Shanghai, China; 7 College of Clinical Medicine, Henan Key Laboratory of Cancer Epigenetics, The First Affiliated Hospital of Henan University of Science and Technology, Luoyang, China; 8 Department of Urology, Guangdong Second Provincial General Hospital, Guangzhou, China; 9 Department of Urology, The Affiliated Guangdong Second Provincial General Hospital of Jinan University, Guangzhou, China; 10 State Key Laboratory of Bioactive Molecules and Druggability Assessment, MOE Key Laboratory of Tumor Molecular Biology and Institute of Precision Cancer Medicine and Pathology, School of Medicine, Jinan University, Guangzhou, China; 11 Department of General Surgery, The First Affiliated Hospital of Jinan University, Guangzhou, China

**Keywords:** Breast cancer, Lung metastasis, Epithelial‒mesenchymal transition, Tyrosine dephosphorylation, JAK2‒YAP pathway, Cancer, Cell biology

## Abstract

Lung metastasis is the primary cause of breast cancer-related mortality. Protein tyrosine phosphatases such as PTPRO are important in cancer progression. However, the role and underlying mechanisms of PTPRO in breast cancer lung metastasis are largely unknown. The function of PTPRO in breast cancer metastasis was examined in mice with *ptpro* deficiency driven by the PyMT promoter. The regulatory role of PTPRO in JAK2–YAP activation was tested in cell-based knockdown, overexpression and catalytic-dead mutation assays. Bioinformatics analyses and assays of human cancer specimens and mouse tumour samples were performed to investigate PTPRO-regulated pathways and functions. *Ptpro* deletion in MMTV-*PyMT* transgenic mice led to increased lung metastasis. Bioinformatics analyses and subsequent assays of human breast cancer specimens revealed a reverse correlation between PTPRO expression and JAK2–YAP pathway activity. Both in vitro and in vivo data demonstrated that PTPRO inactivates the JAK2–YAP pathway and diminishes the metastatic ability of breast cancer. Analysis of catalytic-dead PTPRO mutant breast cancer cells confirmed that functional PTPRO is a determinant of the activation of the JAK2–YAP pathway and the suppression of breast cancer metastasis. Data from patient, animal and cell-based models collectively demonstrated that PTPRO suppresses breast cancer lung metastasis by inhibiting JAK2–YAP dephosphorylation. Therefore, strengthening PTPRO or targeting PTPRO-mediated pathways could be potential strategies for inhibiting breast cancer lung metastasis.

**Supplementary Information:**

The online version contains supplementary material available at 10.1038/s41598-025-91341-0.

## Introduction

Breast cancer is the most common malignancy in females worldwide^1^, and lung metastasis is the leading cause of breast cancer-related death^2^. However, the understanding of the mechanism underlying breast cancer lung metastasis is far from complete^2^. Tyrosine phosphorylation plays an important role in cancer progression^3^. The phosphorylation status of specific tyrosine residues is balanced by the activities of protein tyrosine kinases (PTKs) and protein tyrosine phosphatases (PTPs)^3^. While the role of PTKs in cancer metastasis is clear, that of PTPs is less well understood. However, the role of PTPs in cancer metastasis has attracted increasing attention^4^. For example, PTPN18 can subsequently inhibit transforming growth factor and epithelial‒mesenchymal transformation and ultimately suppress breast cancer metastasis by targeting ETS1β signal transduction^5^. We have also shown that PTPRO can suppress breast cancer progression by affecting the phosphorylation status of ERBB2 and related downstream pathways^6^. We further demonstrated that PTPRO serves as a suppressor of ESCC metastasis by dephosphorylating and inhibiting MET-mediated signalling^7^.

YES-associated protein (YAP) is a key component of the Hippo pathway that is involved in cell proliferation, tumorigenesis, chemical resistance, and metastasis^8,9^. The activity of YAP is regulated by several mechanisms, including PI3K- and PDK1-induced nuclear translocation and activation^10,11^. Phosphorylation of YAP at S127 and S397 inhibits its nuclear translocation and consequently promotes its degradation^12^. Conversely, Src-mediated phosphorylation of YAP at Y357 promotes its nuclear translocation^13,14^. Both Src and JAK2 are tyrosine kinases, and JAK2 not only plays an important role in cancer cell apoptosis and metastasis but is activated by PTPRO deletion. Nevertheless, it is unclear whether JAK2 and Src share the same effects on YAP. In this study, we demonstrated that the loss of PTPRO correlated with epithelial‒mesenchymal transition (EMT) and breast cancer lung metastasis. Mechanistically, PTPRO suppresses breast cancer lung metastasis by dephosphorylating JAK2 and inhibiting the JAK2–YAP pathway.

## Results

### PTPRO inhibits BC cell lung metastasis

**Fig. 1:**
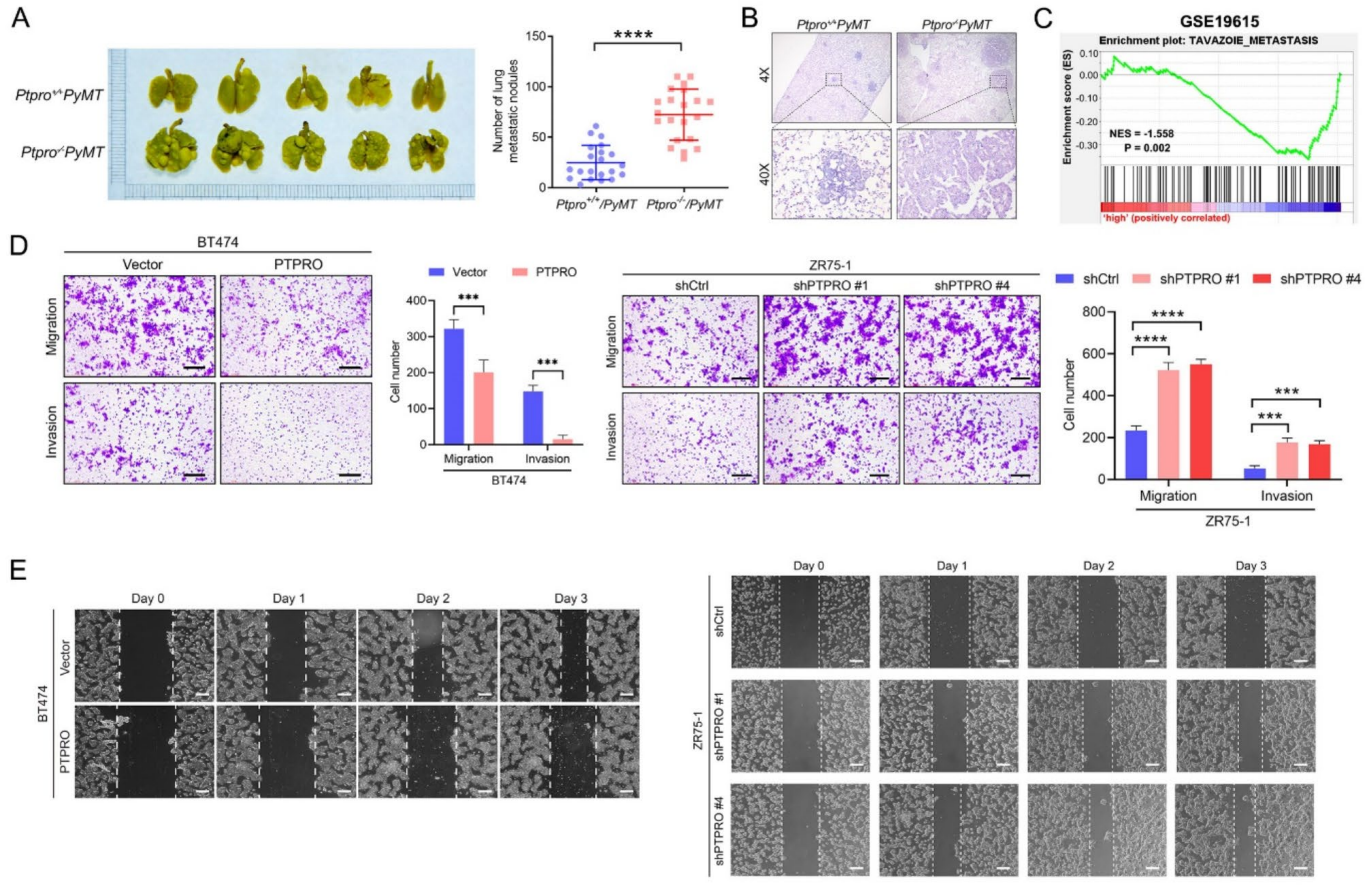
PTPRO suppresses the metastatic potential of breast cancer. (**A**) Representative images of lung metastasis in vivo and statistical analysis of the number of metastatic foci in the indicated groups of mice (*n* = 21). (**B**) The metastatic foci in the lungs were examined using HE staining. (**C**) PTPRO expression inversely correlates with the metastasis-related gene signature according to a gene set enrichment analysis plot. (GSE19615; *P* = 0.002; NES, normalized enrichment score). (**D**–**E**) The migration and invasion abilities of BT474-overexpressing PTPRO cells and ZR75-1-knockdown PTPRO cells were measured by Transwell and scratch healing assays, respectively (left). Quantitative analyses of cell migration and invasion (right). The error bars indicate the SEM. **P < 0.01, ***P < 0.001, ****P < 0.0001 by Student’s *t* test (**A**) or one-way ANOVA with post hoc intergroup comparisons (**D**).

To determine whether PTPRO plays a role in BC cell lung metastasis, we established a mouse mammary tumour model (*Ptpro*^*+/+*^*PyMT* and *Ptpro*^*−/−*^*PyMT)*, and the nodules in the lungs were examined when the size of the tumour reached approximately 1200 mm^3^. The number of nodules in the lungs was greater in the *Ptpro*^*−/−*^*PyMT* mice three times than in the control (Fig. 1A), as evidenced by HE staining (Fig. 1B). This finding is consistent with the metastasis-related gene signature obtained from gene set enrichment analysis (GSEA) that is enriched in PTPRO-low BC patients (Fig. 1C) (GSE19615)^15^. The suppressive role of PTPRO in metastasis has also been confirmed in two BC cell lines, BT474 and ZR75-1, which express low and high levels of PTPRO, respectively. Furthermore, using cell lines with either stable PTPRO expression or PTPRO knockdown (Supplementary Fig. S1), we demonstrated the effects of PTPRO on BC cell migration and invasion with Transwell and scratch healing assays, respectively. Figure 1D and E show that both migration and invasion were enhanced when PTPRO was knocked down in ZR75-1 cells and suppressed when PTPRO was overexpressed in BT474 cells.

### PTPRO inhibits BC cell lung metastasis by suppressing EMT

**Fig. 2:**
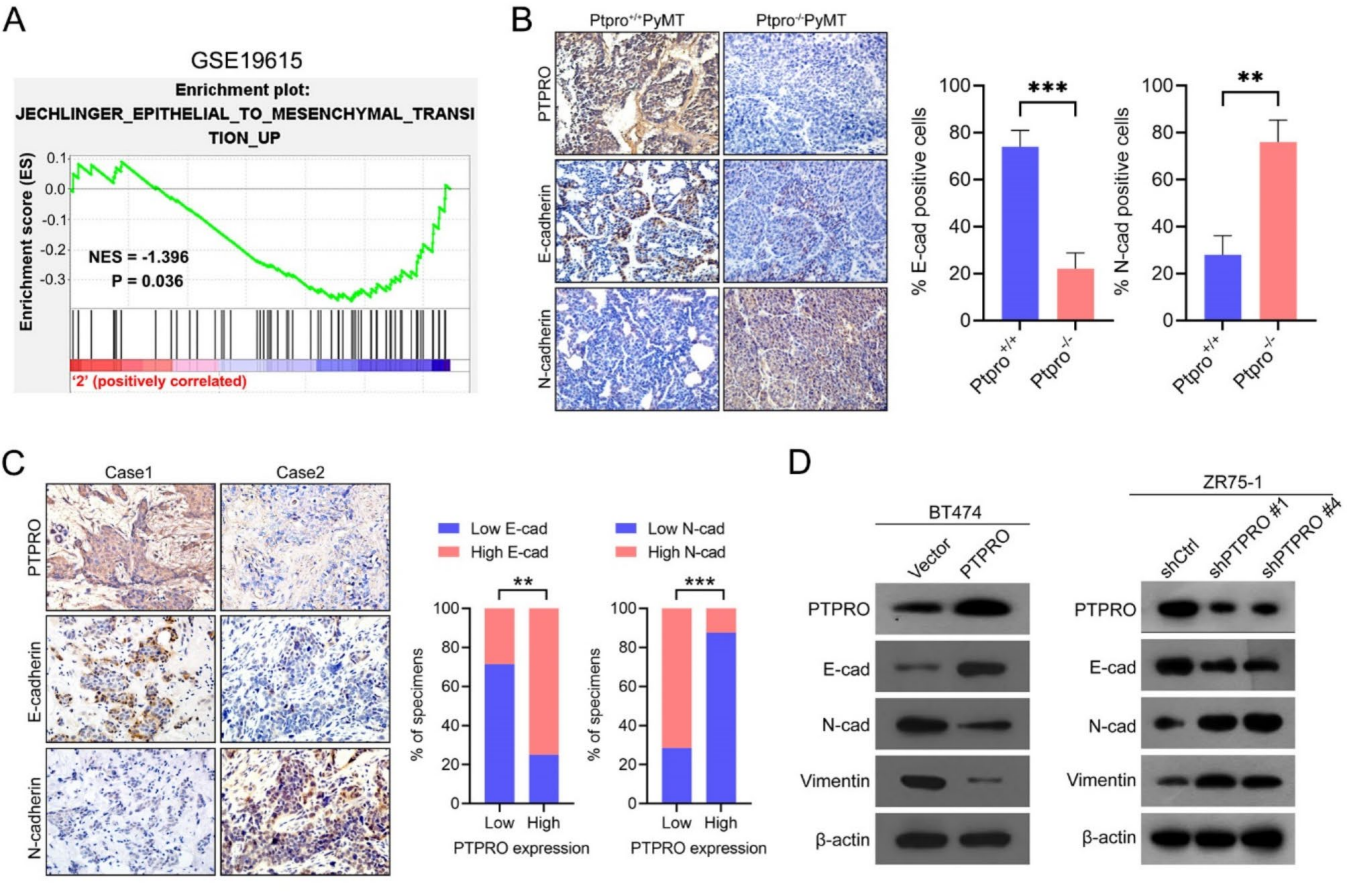
PTPRO suppresses EMT in breast cancer. (**A**) PTPRO expression inversely correlates with the EMT-related gene signature according to a gene set enrichment analysis plot (GSE19615; *P* = 0.036; NES, normalized enrichment score). (**B**) Representative images of immunohistochemistry detection of *Ptpro*, E-cad and N-cad in the tumours of *Ptpro*^−/−^ *PyMT* mice (left). The bar charts show the quantification of the staining (right). (**C**) Representative immunohistochemistry images of PTPRO, E-cad and N-cad in the same tumours from two primary human breast cancer samples (left). Percentages of samples showing E-cad and N-cad expression relative to the level of PTPRO in 15 human breast cancer samples (right). (**D**) Western blotting was used to detect the expression levels of EMT-related markers in BT474 cells overexpressing PTPRO and ZR75-1-knockdown PTPRO cells, with β-actin as the internal reference. The error bars indicate the SEM. **P < 0.01, ***P < 0.001 by Student’s *t* test (**B**) or Spearman’s rank test (**C**).

We next examined the level of PTPRO in breast tumours in GEO datasets, which revealed that PTPRO was highly correlated with multiple EMT-related markers (Fig. 2A) (GSE19615)^15^. IHC staining also showed that the levels of PTPRO were positively and negatively correlated with those of E-cad and N-cad, respectively, in both animal model and human breast cancer samples (Fig. 2B and C). The results from WB assays revealed that in BT474 cells, PTPRO overexpression upregulated E-cadherin and downregulated vimentin and N-cadherin, whereas PTPRO knockdown had the opposite effects (Fig. 2D). Taken together, these results demonstrated the suppressive role of PTPRO in EMT.

### PTPRO suppresses BC lung metastasis by targeting the JAK2–YAP axis

**Fig. 3:**
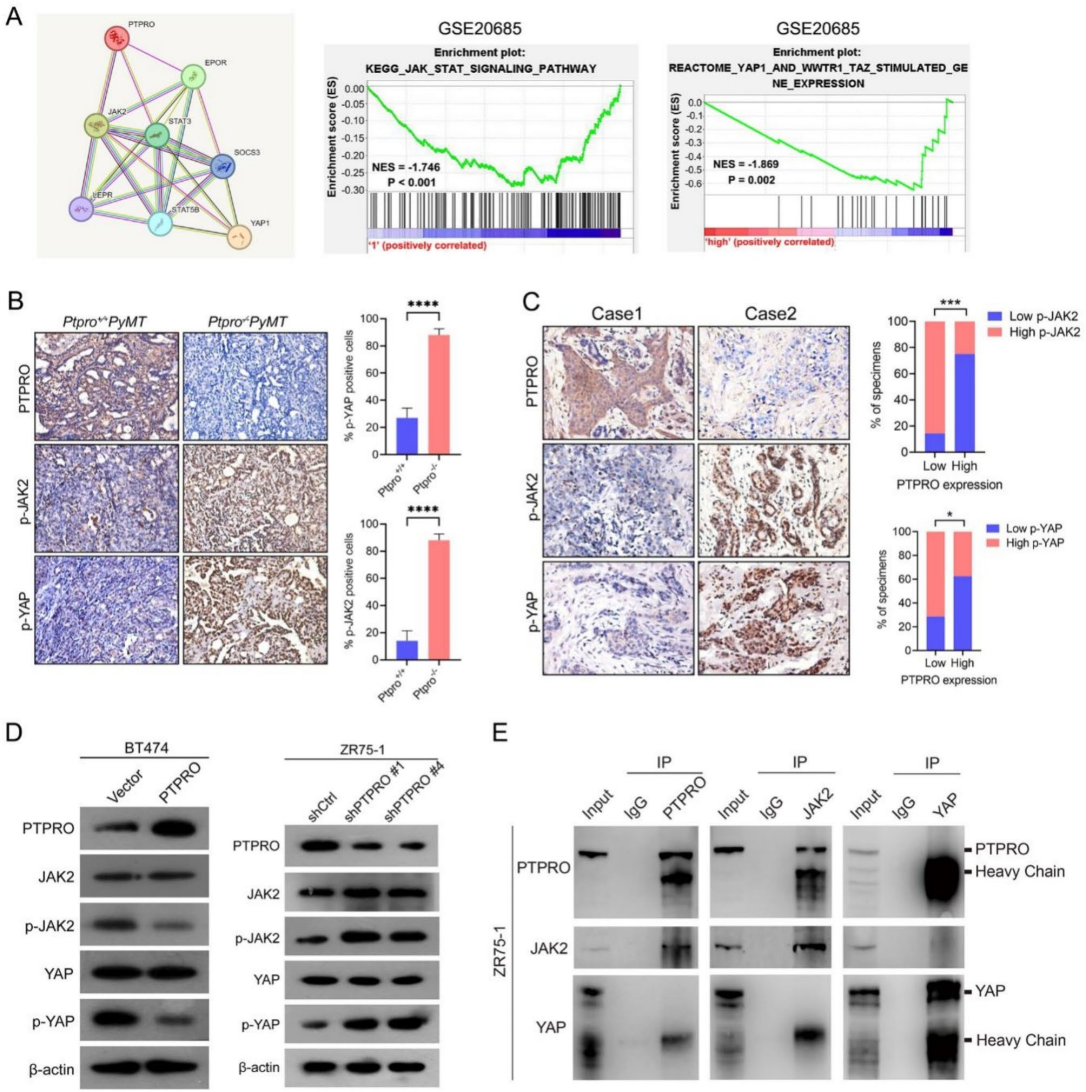
PTPRO inactivates the JAK2–YAP pathway. (**A**) Prediction of the protein interaction network downstream of PTPRO. A protein‒protein interaction network illustrating the interactions between PTPRO and seven PTPRO-associated proteins was constructed using the STING interaction group database (networkhttps://stringdb.org/cgi/network). According to the gene set enrichment analysis, the expression of PTPRO was negatively correlated with that of proteins in the JAK2/STAT and YAP/TAZ pathways (GSE20685; *P* = 0.002, *P* < 0.001, respectively; NES, normalized enrichment score). (**B**) Representative images of immunohistochemistry detection of PTPRO, p-JAK2 (Y1007/Y1008) and p-YAP (Y357) in *Ptpro*^*+/+*^*PyMT* and *Ptpro*^*−/−*^ *PyMT* mouse mammary tumours (left). Bar charts show the quantification of the staining (right). (**C**) Representative immunohistochemistry images of PTPRO, p-JAK2 (Y1007/Y1008) and p-YAP (Y357) in the same tumours from two primary human breast cancer samples (left). Percentage of samples showing p-JAK2 (Y1007/Y1008) and p-YAP (Y357) expression relative to the levels of PTPRO in 15 cases of human breast cancer samples (right). (**D**) Western blotting was used to detect changes in the expression levels of the PTPRO and JAK2–YAP pathways in PTPRO-overexpressing BT474 cells and PTPRO-knockdown ZR75-1 cells. β-actin was used as the internal reference. (**E**) Co-immunoprecipitation showed interaction between PTPRO, JAK2, and YAP in the ZR75-1 cell line with endogenous high expression of PTPRO. Immunoprecipitation (IP) was performed using anti-PTPRO, anti-JAK2 or anti-YAP antibodies. The error bars indicate the SEM. *P < 0.05, ***P < 0.001, ****P < 0.0001 by Student’s *t* test (**B**) or Spearman’s rank test (**C**).

**Fig. 4:**
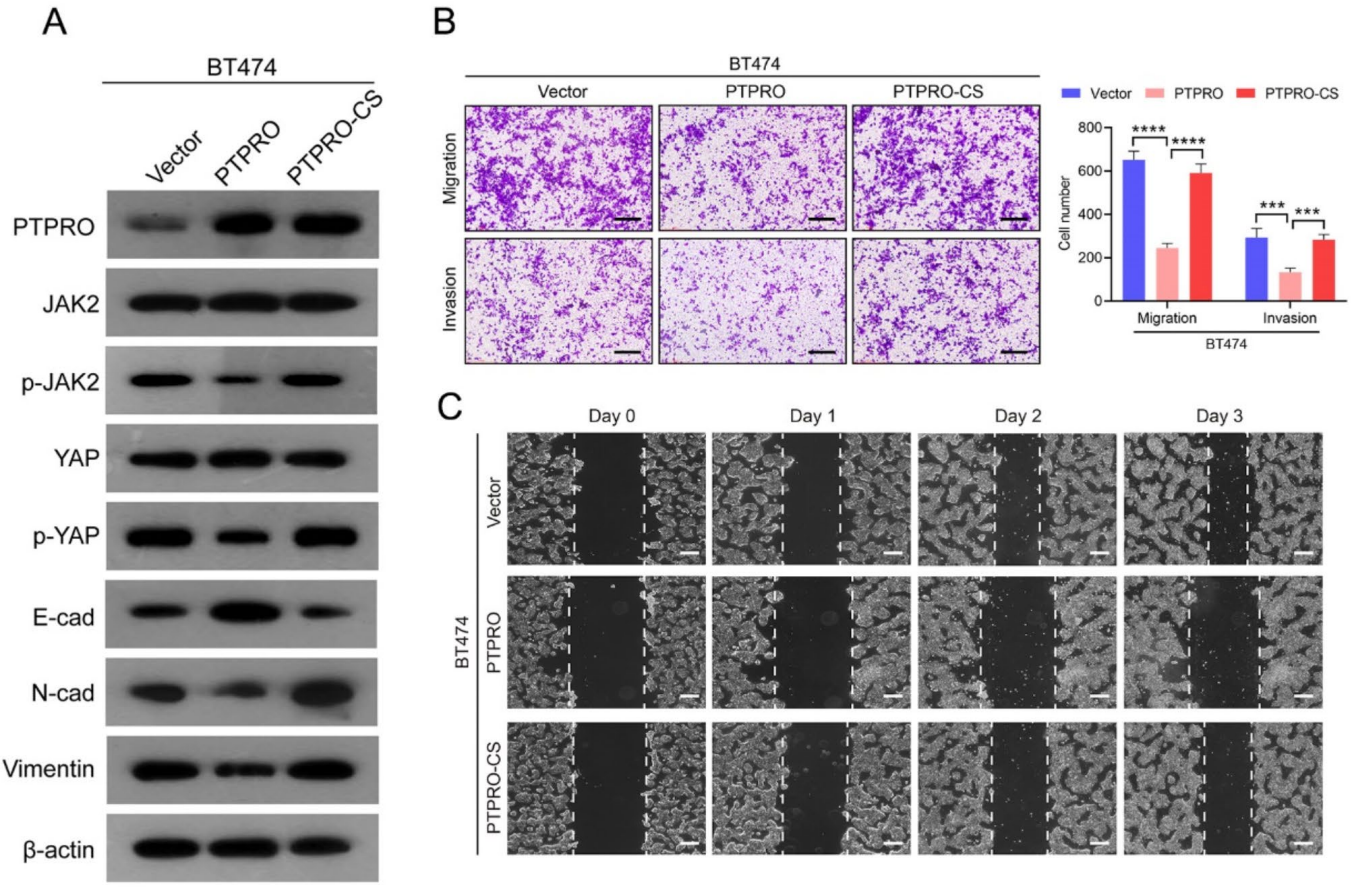
PTPRO phosphatase activity is a determinant of JAK2–YAP pathway activation and the suppression of breast cancer metastasis. (**A**) Proteins extracted from BT474 cells transfected with vector, PTPRO, or PTPRO CS were analysed via Western blotting with antibodies against PTPRO, p-JAK2, p-YAP, E-cad, N-cad, and Vimentin; β-actin was used as an internal reference. (**B**) Representative images of BT474 cell migration and invasion (vector vs. PTPRO vs. PTPRO CS) (left). Scale bars: 100 μm. Quantitative analyses of cell migration and invasion (right). (**C**) Representative image of BT474 cell scratch healing (vector vs. PTPRO vs. PTPRO CS). The error bars indicate the SEM. ***P < 0.001, ****P < 0.0001 by one-way ANOVA with post hoc intergroup comparisons.

To understand the underlying molecular mechanism by which PTPRO inhibits EMT and metastasis, we first conducted protein‒protein interaction (PPI) analysis and identified 7 potential PTPRO-interacting candidates, including JAK2, STAT3, STAT5B, YAP, EPOR, SOCS3, and LERR (Fig. 3A). GSEA revealed that PTPRO is negatively correlated with the JAK2/STAT kinase signalling pathway and transcriptional coactivators of the YAP/TAZ signalling pathway (Fig. 3A)(GSE20685)^16^. Since PTPRO can suppress tumour progression and metastasis by targeting STAT3 and JAK2^17^, we focused our research on the JAK2/YAP pathway. *Ptpro* deficiency was correlated with JAK2/YAP activation in both *PyMT* and human breast tumours (Fig. 3B,C). On the other hand, the levels of phosphorylated JAK2 and YAP were increased and decreased in PTPRO-knockdown and PTPRO-overexpressing cells, respectively (Fig. 3D). Furthermore, Co-IP experiments to examine whether PTPRO interacts with JAK2 and YAP. The results demonstrated that in the ZR75-1 cell line with endogenous high expression of PTPRO, PTPRO interacts with JAK2, but there is no interaction between PTPRO and YAP, nor between JAK2 and YAP (Fig. 3E). To test whether the phosphatase activity of PTPRO is required for JAK2–YAP signalling pathway activity, we compared the effects of wild-type PTPRO and a catalytic site-mutant PTPRO (CS) on BT474 cell migration and invasion. Figure 4A shows that the JAK2/YAP signalling pathway and EMT were repressed by wild-type PTPRO but not by CS. Furthermore, the suppressive effect of PTPRO on BC cell migration and invasion was significantly reduced in the CS cells (Fig. 4B,C). Taken together, these data suggest that PTPRO-mediated dephosphorylation of JAK2 and YAP is likely involved in PTPRO-mediated inhibition of BC lung metastasis.

## Discussion

Despite great advances in treatment, patients with metastatic breast cancer still have a much worse prognosis than those with primary breast cancer^18^. To better understand the mechanism of breast cancer lung metastasis, we first compared BC cell migration and invasion in mice with either wild-type or *Ptpro*-null tumours and identified the suppressive role of PTPRO in breast cancer lung metastasis. We then demonstrated that PTPRO-mediated simultaneous dephosphorylation of JAK2 and YAP plays an essential role in EMT repression. To our knowledge, this is the first study to demonstrate that PTPRO inhibits BC cell metastasis by targeting the JAK2–YAP axis. Since PTPRO expression is inversely correlated with cancer cell metastasis, increasing PTPRO expression could be a potential therapeutic strategy to prevent BC metastasis.

Aberrant expression of protein tyrosine phosphatases (PTPs) has been implicated in various cancers, exerting either tumour-suppressive functions through dephosphorylation of oncogenic proteins or promoting cancer development and progression by positively regulating other signalling pathways^3,19,20^. Previous studies have highlighted the roles of PRL2 and PTPN1 in promoting breast cancer metastasis through the activation of extracellular signal-regulated kinase 1/2 and the upregulation of the expression of vitamin D receptors, respectively^21,22^. Notably, both PRL2 and PTPN1 appeared to be cancer-promoting factors. Conversely, our investigation revealed that PTPRO has an inhibitory effect on breast cancer lung metastasis. Consistent with this, in previous research, we demonstrated that PTPRO impedes the occurrence and progression of Her2-positive breast cancer by dephosphorylating ERBB2^6^. Here, we demonstrated that PTPRO suppresses breast cancer lung metastasis by targeting the JAK2–YAP axis.

PTPRO may exert its anticancer effect by regulating multiple signalling pathways in a cancer type-specific manner^23–25^. For example, PTPRO inhibits colorectal cancer liver metastasis by dephosphorylating AKT and MAPK^26^. We have demonstrated that PTPRO inhibits oesophageal cancer lymph node metastasis by dephosphorylating MET^7^. As an extension of our previously established conceptual framework, in which PTPRO downregulates tyrosine phosphorylation of multiple oncogenic pathways in breast carcinogenesis, we experimentally confirmed the functional association between PTPRO phosphatase activity and the JAK2–YAP axis. We also demonstrated the essentiality of its catalytic activity in suppressing breast cancer lung metastasis, as evidenced by the observation that PTPRO with an active site mutation failed not only to dephosphorylate JAK2 and YAP but also to inhibit breast cancer lung metastasis.

The Hippo–YAP pathway was first identified in *Drosophila melanogaster* and governs tissue growth and organ size^10,11^. Its core components include two serine/threonine kinases, namely, Hippo and Warts, alongside the transcriptional coactivator YAP^10,11^. The function of YAP depends on its subcellular localization, with its nuclear‒cytoplasmic shuttling contingent upon its phosphorylation status^10,11^. Notably, phosphorylation at S127 and S397 hinders the nuclear translocation of YAP, whereas phosphorylation of Y357 promotes its nuclear localization. Consistent with this, YAP phosphorylation at Y357 in MCF10A cells has been shown to induce EMT, a process that has been implicated in carcinoma progression and metastasis^27^. Furthermore, direct interaction between the PPxY motif of PTPN14 and the WW domain of YAP is essential for YAP phosphorylation at Y357^27^. Therefore, given that it lacks a PPxY motif for direct YAP binding, PTPRO should be unable to directly dephosphorylate YAP. However, PTPRO is capable of regulating the tyrosine kinase activity of JAK2. These findings are also supported by the results from protein‒protein interaction network analysis, which revealed that PTPRO can modulate YAP phosphorylation by regulating JAK2.

In summary, we have demonstrated through both in vivo and in vitro experiments that PTPRO suppresses breast cancer metastasis. Mechanistically, PTPRO inhibits EMT through the dephosphorylation of JAK2 and YAP. Given the critical function of PTPRO in inhibiting metastasis, targeting PTPRO could be a potential therapeutic strategy for preventing breast cancer lung metastasis.

## Materials and methods

### Human samples

Human breast tissue samples were taken from breast cancer patients receiving surgery in the Cancer Hospital of Shantou University School of Medicine from 2010 to 2013. All patients underwent surgery as the main treatment, followed by adjuvant radiotherapy, chemotherapy, or hormone therapy. The Institutional Review Committee and Ethics Committee of the Cancer Hospital of Shantou University School of Medicine, Shantou, China, reviewed and approved the clinical study protocol for this study (IRB Serial No. # 04–070). According to the principles expressed in the Declaration of Helsinki, the patient’s written informed consent was obtained.

### Animals

The *Ptpro*^*−/−*^ mice, gifts from Dr Bixby, University of Miami, and FVB/N mice carrying the *PyMT* gene (*MMTV-PyMT*) were obtained from Jackson Laboratories. Animals were housed in conventional or pathogen-free conditions, where appropriate, at the Animal Center of Shantou University Medical College in compliance with the Institutional Animal Care and Use Committee (IACUC) regulations (SUMC2014-148). All animal experiments were performed according to protocols approved by the Animal Care and Use Committee of Shantou University Medical College. All mice were raised and bred at the Animal Center of Shantou University School of Medicine. All experiments were performed in accordance with relevant guidelines and regulations. Hybridize *Ptpro* ^−/−^ mice with wild-type FVB/N mice for 10 generations to obtain 99.90% FVB/N background *Ptpro* ^−/−^ mice, and pair their female offspring with *MMTV-PyMT* transgenic male mice with FVB strain background. Group assignment was based on genotypes, and no randomization was involved. The sample size of 21 mice per group was calculated based on the software G*Power 3.1 (significance = 0.05; power = 0.8, and effect size = 0.8). Age-matched *Ptpro*^*+/+*^*/MMTV-PyMT* and *Ptpro*^*−/−*^*/MMTV-PyMT* virgin mice with 99.95% FVB/N genetic background (11 generation backcross to FVB/N strain) were used for further experiments. Littermates with both genotypes were used in the same phenotypic alterations whenever it was possible. collected for morphologic and biochemical analyses. No blinding was done. Tumours were measured 2 ~ 3 times per week, and mice were executed for tissue collection using CO_2_ euthanasia when the tumour volume reached 1200 mm^3^. CO_2_ was injected into the euthanasia box for 30 s. Mice were placed in the box and then CO_2_ was injected into the box for 5 min to ensure that the mice did not move, and then the animals were observed for 2 min to ensure that they were dead. At the experimental endpoint, tumors and lung tissues were harvested and fixed with 4% PFA for paraffin-embedded section. Whole mouse lung tissues were fixed in Bouins solution, the metastasis nodules in lung tissues were analyzed by hematoxylin and eosin (HE) staining methods. Their genotypes were identified by PCR analyses of tail DNA samples. The primers of mice genotypes analysis are as follows:

**Table:** 

*Ptpro*	Fw: 5’-AAACCTTAAACTCCTGATCCTCCTGCCTCC-3’ Targeted: 5’-GCCTTCTATCGCCTTCTTGACGAGTTCTTC-3’ Rev: 5’-CACTGAATCAAAATGTCCCACCCATGTTTC-3’

All methods are reported by ARRIVE guidelines (https://arriveguidelines.org) for the reporting of animal experiments.

### Bioinformatics analysis

Gene Set Enrichment Analyses (GSEA) were performed using the GSEA software (version 4.2.3) (http://www.broadinstitute.org/gsea/index.jsp). The significance of enrichments is presented by normalized enrichment scores in normal *p*-values.

### Cell culture

Human breast cancer cell lines were obtained from the American Type Culture Collection (ATCC). BT474 and ZR75-1 cells were grown in DMEM/F12 (GIBCO/Invitrogen, Carlsbad, CA) containing 10% fetal bovine serum (GIBCO/Invitrogen, Carlsbad, CA). All cells were maintained at 37 °C in an incubator containing 5% CO_2_. All cell lines have been authenticated using short tandem repeat DNA profiling (Beijing Microread Genetics Co., Ltd., Beijing, China).

### Plasmid constructs and transfection

The full-length coding sequences of PTPRO-WT were cloned into pCR3.1-HA and pCDNA3.1-3xFlag expression vectors, respectively. The catalytic site mutation form of PTPRO is C1136S mutation (CS), which is produced through site directed mutagenesis using the QuickChange Site Directed Mutagenesis kit (Stratagene) according to the manufacturer’s instructions. PTPRO-CS is a mutation of cysteine ​​(Cys/C) at position 1136 of the PTPRO gene to serine (Ser/S), hence causing PTPRO to lose its phosphatase function. Plasmid pGIPZ shRNA vectors targeting human PTPRO (pGIPZ-shPTPRO #1, V2LHS_226171; pGIPZ-shPTPRO #4, V2LHS_227542) and non-targeting pGIPZ control vector (pGIPZ-shCtrl) were obtained from Open Biosystems (Huntsville, AL, USA). To induce overexpression of PTPRO and PTPRO-CS in BT474, the cells were stably transfected with the plasmid DNA of pCR3.1-PTPRO, pCR3.1-PTPRO-CS or the control plasmid pCR3.1. For inhibition of PTPRO expression in ZR75-1 cells, the cells were stably transfected with the plasmid DNA of pGIPZ-shPTPRO #1, pGIPZ-shPTPRO #4 or pGIPZ-shCtrl. All transfections were performed with Lipofectamine 8000 (beyotime) according to the manufacturer’s instructions.

### Quantitative real-time PCR (RT-qPCR)

Total RNA was extracted from the cells using NcmSpin Cell/Tissue Total RNA Kit (NCM Biotech cat. No. M5105). Complementary DNA synthesis was performed using a BcaBest RNA PCR kit from Takara Bio, Inc. according to the manufacturer’s protocols. qPCR was performed using an CFX Connect™ Thermal Cycler Real-Time PCR Detection system (Bio-Rad Laboratories, Inc.) with Realtime PCR Master Mix (MagicSYBR Mixture). The thermocycling conditions used for qPCR were as follows: 95˚C for 30 s, followed by 40 cycles at 95˚C for 5 s and 60˚C for 30 s, in a total volume of 20 µl. Relative mRNA expression levels were assessed using the 2-ΔΔCq method. β-actin was used as the exogenous control. The PCR primer sequences used were as follows:

**Table:** 

PTPRO	Fw: 5’-TGGCTGCCAGGAATGTGTTA-3’ Rev: 5’-TAAGGGGCAGTTCTGTGCTG-3’
β-actin	Fw: 5’-TGCACCACCAACTGCTTAGC-3’ Rev: 5’-GGCATGGACTGTGGTCATGAG-3’

### Western blot analysis

All cell lysates were prepared using RIPA lysis (Beyotime) and intact protease inhibitors (Beyotime). The protein concentration was determined using a BCA protein assay kit (Pierce; Thermo Fisher Scientific, Inc.). The samples (20 µg/line) were separated by 10% SDS-PAGE and transferred to polyvinylidene fluoride membranes (Roche Diagnostics). The membranes were blocked using 5% BSA (bovine serum albumin) in TBST, followed by incubation with the primary antibodies at 4˚C overnight and then HRP-labeled secondary antibodies at room temperature for an hour (1: 5,000, Cat. No. 61‑6520, HRP‑labeled, Thermo Fisher Scientific, Inc). The immunolabeled proteins were detected using an ECL detection system (NCM Biotech Cat. No P10300). The primary antibodies used were as follows: β-actin (1:1,0000, Cat. No. 66009-1-Ig; proteintech), E-cadherin (1:20000, Cat. No. 20874-1-AP, proteintech), N-cadherin (1:5000, Cat. No. 22018-1-AP, proteintech), vimentin (1:5000, Cat. No. 10366-1-AP, proteintech), PTPRO (1:500, Cat. No. 67000-1-Ig, proteintech), JAK2(1:1000, Cat. No. ab108596, abcam), p-JAK2(1:1000, Cat. No. ab32101, abcam), YAP (1:1000, Cat. No. 14074, Cell Signaling technology) and p-YAP (1:1000, Cat. No. ab62751, abcam). The immunoreactive bands were visualized with Super Signal West Pico Chemiluminescent Substrate (Thermo Scientific) using X-ray film (FUJIFILM).

### Scratch healing assays

BT474 and ZR75-1 cells were seeded in six-well plates and cultured at 37˚C overnight in DMEM/F12 medium. A wound was then created by manually scraping the cell monolayer with a 10 µl pipette tip. The cultures were washed twice with PBS to remove floating cells. The cells were then incubated at 37˚C in DMEM/F12 (Gibco; Thermo Fisher Scientific, Inc.) supplemented with 2% FBS (Solarbio, Beijing Solarbio Science & Technology). Cell migration into the wound was assessed at five preselected time points (0, 12,24,48 and 72 h) in six randomly selected fields of view for each condition and time point. The distance traveled by the cells was determined by measuring the wound width at different time points. The formula of calculation is as follows: cell migration rate = (scratch area before treatment – scratch area after treatment) / (scratch area before treatment) × 100%.

### Transwell migration and invasion assay

BT474 and ZR75-1 cells (1 × 10^5^) were added on the top side of polycarbonate Transwell filters (without Matrigel for Transwell migration assays) or plated on the top side of polycarbonate Transwell filter coated with Matrigel at 37˚C for an hour (for Transwell invasion assay) in the upper chamber of the QCM™ 24‑Well Cell Invasion Assay (Cell Biolabs, Inc.) plates. For Transwell migration and invasion assays, cells were suspended in 200 µl of DMEM/F12 containing 2% FBS and 750 µl of DMEM/F12 containing 20% FBS was used in the lower chamber. The cells were incubated at 37˚C for 48 h (migration assay) or 72 h (invasion assay). The cells in the top chambers were then removed using cotton swabs. The migrated and invaded cells on the lower membrane surface were fixed using 4% tissue cell stationary fluid for 20 min at room temperature, airdried, then stained with 5% crystal violet solution (Solarbio, Beijing Solarbio Science & Technology) at room temperature for 20 min.

### Immunohistochemistry

Immunofluorescence was performed as described previously^28,29^. Four-µm-thick sections from formalin-fixed paraffin-embedded clinical specimens and mice mammary tumors were processed according to standard Immunohistochemistry protocols and stained with antibodies against E-cadherin (1:200, Cat. No. 20874-1-AP, proteintech), N-cadherin (1:200, Cat. No. 22018-1-AP, proteintech), PTPRO (1:200, Cat. No. 67000-1-Ig, proteintech), p-JAK2(1:100, Cat. No. ab32101, Abcam) and p-YAP (1:100, Cat. No. ab62751, Abcam). The percentage of positively stained was scored using the following scales: 0, no staining of cells in any field; 1, ≤ 10%; 2, 11–50%; 3, 51–75%; 4, > 75%. The intensity of staining was scored using the following scales: 1+, weak staining; 2+, moderate staining; 3+, strong staining. Percentage (P) and intensity (I) of nuclear or cytoplasm or membrane expression were multiplied to generate a numerical score (S = P • I). PTPTO expression levels were assessed as positive or negative, according to the cut-off value 2, which is determined by preliminary data derived from normal breast tissue. This requires counting at least 1000 tumor cells with nuclear staining in ten high-power fields (×400). No staining or membrane staining in < 30% of tumor cells. 1+, barely perceptible membrane staining in > 30% of tumor cells or cells only stained in part of the membrane. 2+, weak/moderate complete membrane staining in > 30% of tumor cells. 3+, strong complete membrane staining in > 30% of tumor cells. The immunoreactivity was evaluated by two different pathologists with no prior knowledge of patient data. When the opinions of the two evaluators were different, agreement was then compared and discussed.

### Statistical analysis

Comparisons between two groups were performed with a Student’s *t* test and comparisons among more than two groups were performed with one-way ANOVA with post hoc intergroup comparisons. All bar graphs show the mean ± SEM of at least three independent experiments. *p* value of less than 0.05 was considered statistically significant.

## Electronic supplementary material

Below is the link to the electronic supplementary material.

Supplementary Material 1

## Data Availability

All data obtained and/or analyzed in this study are available from the corresponding author upon reasonable request. The datasets generated and/or analysed during the current study are available in the GEO repository. (https://www.ncbi.nlm.nih.gov/geo/, GSE19615; GSE20685)

## References

[1] Harbeck, N. & Gnant, M. Breast cancer. *Lancet* **389**, 1134–1150. 10.1016/S0140-6736(16)31891-8 (2017). 27865536 10.1016/S0140-6736(16)31891-8

[2] Yousefi, M. et al. Organ-specific metastasis of breast cancer: molecular and cellular mechanisms underlying lung metastasis. *Cell. Oncol. (Dordrecht)*. **41**, 123–140. 10.1007/s13402-018-0376-6 (2018). 10.1007/s13402-018-0376-6 PMC12995240 29568985

[3] Ostman, A., Hellberg, C. & Bohmer, F. D. Protein-tyrosine phosphatases and cancer. *Nat. Rev. Cancer*. **6**, 307–320. 10.1038/nrc1837 (2006). 16557282 10.1038/nrc1837

[4] Welsh, C. L., Pandey, P. & Ahuja, L. G. Protein tyrosine phosphatases: A new paradigm in an old signaling system? *Adv. Cancer Res.* **152**, 263–303. 10.1016/bs.acr.2021.06.001 (2021). 34353440 10.1016/bs.acr.2021.06.001 PMC10544742

[5] Wang, T. et al. Nuclear import of PTPN18 inhibits breast cancer metastasis mediated by MVP and importin beta2. *Cell Death Dis.* **13**10.1038/s41419-022-05167-z (2022). 10.1038/s41419-022-05167-z PMC9388692 35982039

[6] Dong, H. et al. PTPRO represses ERBB2-driven breast oncogenesis by dephosphorylation and endosomal internalization of ERBB2. *Oncogene* **36**, 410–422. 10.1038/onc.2016.213 (2017). 27345410 10.1038/onc.2016.213 PMC5269534

[7] Dong, H. et al. PTPRO suppresses lymph node metastasis of esophageal carcinoma by dephosphorylating MET. *Cancer Lett.* **567**, 216283. 10.1016/j.canlet.2023.216283 (2023). 37331584 10.1016/j.canlet.2023.216283

[8] Piccolo, S., Panciera, T., Contessotto, P. & Cordenonsi, M. YAP/TAZ as master regulators in cancer: modulation, function and therapeutic approaches. *Nat. Cancer*. **4**, 9–26. 10.1038/s43018-022-00473-z (2023). 36564601 10.1038/s43018-022-00473-z PMC7614914

[9] Franklin, J. M., Wu, Z. & Guan, K. L. Insights into recent findings and clinical application of YAP and TAZ in cancer. *Nat. Rev. Cancer*. **23**, 512–525. 10.1038/s41568-023-00579-1 (2023). 37308716 10.1038/s41568-023-00579-1

[10] Zhong, Z., Jiao, Z. & Yu, F. X. The Hippo signaling pathway in development and regeneration. *Cell. Rep.* **43**, 113926. 10.1016/j.celrep.2024.113926 (2024). 38457338 10.1016/j.celrep.2024.113926

[11] Glaviano, A. et al. PI3K/AKT/mTOR signaling transduction pathway and targeted therapies in cancer. *Mol. Cancer*. **22**, 138. 10.1186/s12943-023-01827-6 (2023). 37596643 10.1186/s12943-023-01827-6 PMC10436543

[12] Lv, M. et al. CDK7-YAP-LDHD axis promotes D-lactate elimination and ferroptosis defense to support cancer stem cell-like properties. *Signal. Transduct. Target. Ther.* **8**, 302. 10.1038/s41392-023-01555-9 (2023). 37582812 10.1038/s41392-023-01555-9 PMC10427695

[13] Ma, H. et al. Periostin Promotes Colorectal Tumorigenesis through Integrin-FAK-Src Pathway-Mediated YAP/TAZ Activation. *Cell reports* **30**, 793–806 e796. 10.1016/j.celrep.2019.12.075 (2020). 10.1016/j.celrep.2019.12.075 31968254

[14] Lamar, J. M. et al. SRC tyrosine kinase activates the YAP/TAZ axis and thereby drives tumor growth and metastasis. *J. Biol. Chem.* **294**, 2302–2317. 10.1074/jbc.RA118.004364 (2019). 30559289 10.1074/jbc.RA118.004364 PMC6378979

[15] Li, Y. et al. Amplification of LAPTM4B and YWHAZ contributes to chemotherapy resistance and recurrence of breast cancer. *Nat. Med.* **16**, 214–218. 10.1038/nm.2090 (2010). 20098429 10.1038/nm.2090 PMC2826790

[16] Kao, K. J., Chang, K. M., Hsu, H. C. & Huang, A. T. Correlation of microarray-based breast cancer molecular subtypes and clinical outcomes: implications for treatment optimization. *BMC Cancer*. **11**, 143. 10.1186/1471-2407-11-143 (2011). 21501481 10.1186/1471-2407-11-143 PMC3094326

[17] Dai, Y. et al. Protein tyrosine phosphatase PTPRO represses lung adenocarcinoma progression by inducing mitochondria-dependent apoptosis and restraining tumor metastasis. *Cell. Death Dis.* **15**10.1038/s41419-023-06375-x (2024). 10.1038/s41419-023-06375-x PMC10770368 38182570

[18] Waks, A. G. & Winer, E. P. Breast Cancer treatment: A review. *Jama* **321**, 288–300. 10.1001/jama.2018.19323 (2019). 30667505 10.1001/jama.2018.19323

[19] Yao, Z. et al. Age-related decline in hippocampal tyrosine phosphatase PTPRO is a mechanistic factor in chemotherapy-related cognitive impairment. *JCI Insight*. **8**10.1172/jci.insight.166306 (2023). 10.1172/jci.insight.166306 PMC10443805 37485875

[20] Dong, H. et al. PTPRO-related CD8(+) T-cell signatures predict prognosis and immunotherapy response in patients with breast cancer. *Front. Immunol.* **13**, 947841. 10.3389/fimmu.2022.947841 (2022). 36003382 10.3389/fimmu.2022.947841 PMC9393709

[21] Baumgartner, C. K. et al. The PTPN2/PTPN1 inhibitor ABBV-CLS-484 unleashes potent anti-tumour immunity. *Nature* **622**, 850–862. 10.1038/s41586-023-06575-7 (2023). 37794185 10.1038/s41586-023-06575-7 PMC10599993

[22] Hardy, S., Wong, N. N., Muller, W. J., Park, M. & Tremblay, M. L. Overexpression of the protein tyrosine phosphatase PRL-2 correlates with breast tumor formation and progression. *Cancer Res.* **70**, 8959–8967. 10.1158/0008-5472.CAN-10-2041 (2010). 20841483 10.1158/0008-5472.CAN-10-2041

[23] Xie, F., Dong, H. & Zhang, H. Regulatory functions of protein tyrosine phosphatase receptor type O in immune cells. *Front. Immunol.* **12**, 783370. 10.3389/fimmu.2021.783370 (2021). 34880876 10.3389/fimmu.2021.783370 PMC8645932

[24] You, Y. J., Chen, Y. P., Zheng, X. X., Meltzer, S. J. & Zhang, H. Aberrant methylation of the PTPRO gene in peripheral blood as a potential biomarker in esophageal squamous cell carcinoma patients. *Cancer Lett.* **315**, 138–144. 10.1016/j.canlet.2011.08.032 (2012). 22099875 10.1016/j.canlet.2011.08.032 PMC3248961

[25] Huang, Y. T. et al. PTPRO promoter methylation is predictive of poorer outcome for HER2-positive breast cancer: indication for personalized therapy. *J. Transl Med.* **11**, 245. 10.1186/1479-5876-11-245 (2013). 24090193 10.1186/1479-5876-11-245 PMC3852714

[26] Dai, W. et al. PTPRO represses colorectal cancer tumorigenesis and progression by reprogramming fatty acid metabolism. *Cancer Commun. (Lond)*. **42**, 848–867. 10.1002/cac2.12341 (2022). 35904817 10.1002/cac2.12341 PMC9456702

[27] Liu, X. et al. PTPN14 interacts with and negatively regulates the oncogenic function of YAP. *Oncogene* **32**, 1266–1273. 10.1038/onc.2012.147 (2013). 22525271 10.1038/onc.2012.147 PMC4402938

[28] Ye, G. et al. The FAP alpha -activated prodrug Z-GP-DAVLBH inhibits the growth and pulmonary metastasis of osteosarcoma cells by suppressing the AXL pathway. *Acta Pharm. Sin B*. **12**, 1288–1304. 10.1016/j.apsb.2021.08.015 (2022). 35530139 10.1016/j.apsb.2021.08.015 PMC9072247

[29] Shi, C. et al. ATP-adenosine axis regulation combined with microneedle assisted photoimmunotherapy to boost the immunotherapy efficiency. *J. Control Release*. **367**, 1–12. 10.1016/j.jconrel.2024.01.035 (2024). 38244844 10.1016/j.jconrel.2024.01.035

