# Supplementary figures and images for: PTPRO represses breast cancer lung metastasis by inhibiting the JAK2-YAP axis
Source: Sci Rep. 2025 Feb 27;15:7065. doi: 10.1038/s41598-025-91341-0 (PMC11868365; doi:10.1038/s41598-025-91341-0)

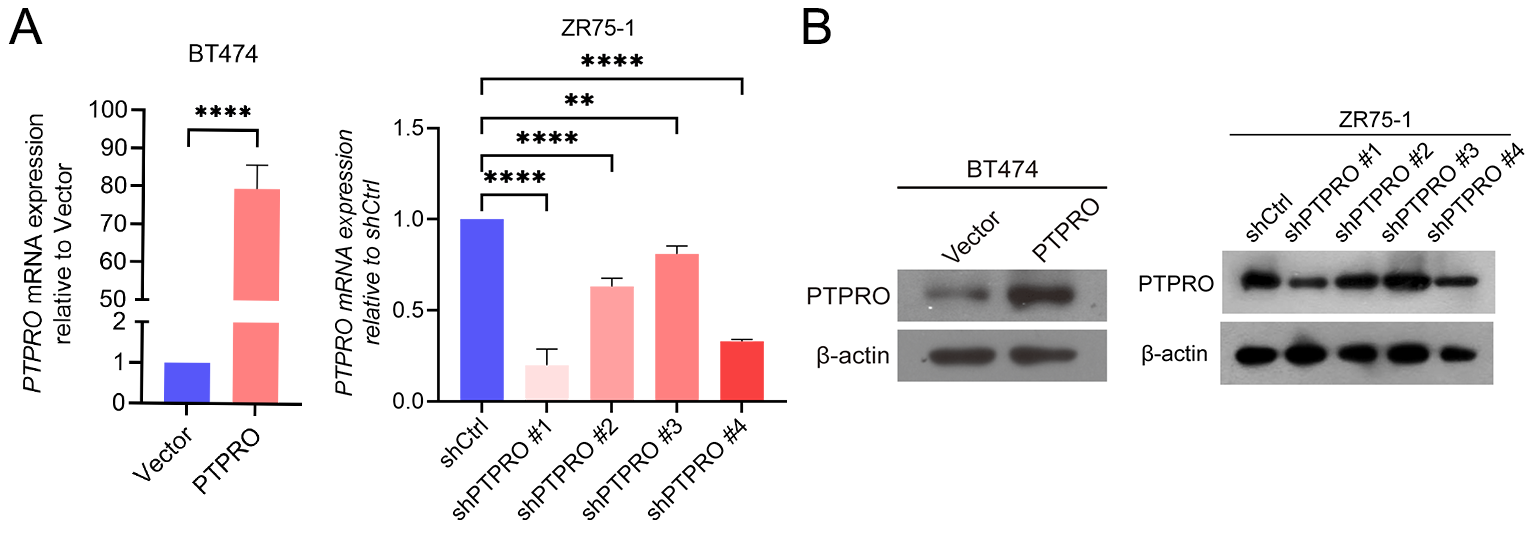

Supplement: Supplementary file 1 — Supplementary Material 1 [file 41598_2025_91341_MOESM1_ESM.tif]
